# Adolescent Experiences of Violence Victimizations Among Minors Who Exchange Sex/Experience Minor Sex Trafficking

**DOI:** 10.1177/08862605211021967

**Published:** 2021-06-30

**Authors:** Hannabeth Franchino-Olsen, Sandra L. Martin, Carolyn T. Halpern, John S. Preisser, Catherine Zimmer, Meghan Shanahan

**Affiliations:** 1 University of North Carolina at Chapel Hill, NC, USA

**Keywords:** minor, sex trafficking, sex exchange, adolescence, violence

## Abstract

This work investigates the associations between experiences of domestic minor sex trafficking and adolescent interpersonal violence victimizations, including intimate partner violence (IPV) and community violence. Abuse and violence in childhood are commonly proposed as risk factors for domestic minor sex trafficking. However, less is known about how interpersonal violence victimizations in adolescence connect to domestic minor sex trafficking experiences. The poly-victimization framework provides a means to understand domestic minor sex trafficking as a type of violence amid a web of additional interconnected violence victimizations. Efforts to better understand the interpersonal violence experienced by survivors of domestic minor sex trafficking are valuable in contextualizing trafficking experiences for adolescents. Data from The National Longitudinal Study of Adolescent to Adult Health, a population-based sample of adolescents in the United States (*n* = 12,605) were used to examine experiences of domestic minor sex trafficking for minor respondents, as measured through questions about exchanging sex for money or drugs. A multivariable logistic regression model was used to estimate the associations between domestic minor sex trafficking and IPV or community violence, while controlling for demographic variables and adolescent risk behaviors. Minors who experience community violence had significantly greater odds of having exchanged sex (aOR: 1.86; 95% CI: 1.32 -2.64). However, IPV was not significantly associated with minors’ experiences of sex exchange (aOR: 1.14; 95% CI: 0.85 -1.54). Alcohol or drug use (aOR: 1.87; 95% CI: 1.32 -2.65) and having run away (aOR: 2.04; 95% CI: 1.53 -2.72) were also significantly associated with minor sex exchange. As experiences of domestic minor sex trafficking were significantly associated with community violence victimizations, prevention and intervention efforts targeting youth at high risk for or survivors of domestic minor sex trafficking should consider how community violence victimizations impact these adolescent populations, and programming/messaging should be adjusted to account for these additional violence victimizations.

## Introduction

Minor sex trafficking in the United States (U.S.) is frequently referred to as domestic minor sex trafficking (DMST), and it is defined as any minor (under age 18) who is a U.S. citizen or legal resident and who is involved in a commercial sex act (Kotrla, 2010). Unlike sex trafficking of an adult, DMST does not require the presence of force, fraud, or coercion for the commercial sex act to be considered trafficking, as minors cannot legally consent to engage in commercial sex exchanges (Kotrla, 2010; Victims of Trafficking and Violence Protection Act, 2000). DMST, a form of commercial sexual exploitation of children (CSEC), includes all forms of sexual involvement of minors in underground economies (Greenbaum, 2014). The terms DMST and CSEC are often used synonymously or interchangeably, but for this study DMST will be used as the focus is on commercial sex acts and excludes stripping and pornography of minors, which are also captured in CSEC (Greenbaum, 2014).

Due to the relatively hidden nature of the issue, statistics capturing the prevalence of DMST or size of the population experiencing DMST are difficult to estimate (Stransky & Finkelhor, 2008). Currently, no credible prevalence or count estimates for DMST exist and recent scholarship has cautioned against continuing to cite current flawed estimates (Franchino-Olsen et al., 2020; National Academies of Sciences Engineering and Medicine, 2020). However, DMST has been noted as occurring across all fifty states and not being isolated to a single demographic group. Further, the antihuman trafficking field is rapidly adapting and applying emerging methodologies to generate improved estimates of human trafficking, including estimates of DMST (Franchino-Olsen et al., 2020; National Academies of Sciences Engineering and Medicine, 2020).

Numerous studies have examined the risk factors and correlates that create vulnerability for DMST (Choi, 2015; Franchino-Olsen, 2019; IOM & NRC, 2013). A history of abuse and violence in childhood and adolescence—including childhood abuse or maltreatment; rape or adolescent sexual victimization; or dating violence—are commonly cited risk factors for DMST (Franchino-Olsen, 2019; U.S. Department of Health and Human Services Administration for Children Youth and Families [ACYF], 2013). Additionally, witnessing family violence or intimate partner violence (IPV) between caregivers may increase a minor’s risk of subsequent DMST experiences (Franchino-Olsen, 2019). Studies using data from the National Longitudinal Study of Adolescent to Adult Health (Add Health) found significant correlates between DMST or young adult (age 18 or older) sex exchange and drug use and binge drinking, shoplifting, running away, homelessness, history of abuse or neglect, and experiences of depression (Edwards et al., 2006; Kaestle, 2012; Ulloa et al., 2016).

Females are often assumed to represent the vast majority of DMST victims/survivors, and much of the research in the previous two decades has focused on females’ victimizations or used samples in which cisgender females are the most common gender of victim/survivor (Robert & Willis, 2013). More attention needs to be given to the nature of victims/survivors who are cisgender males or who represent a gender minority (including transgender, gender nonconforming, or gender nonbinary), especially as some studies have suggested that the latter experience a disproportionate degree of DMST (Clawson et al., 2009; Robert & Willis, 2013; Roe-Sepowitz et al., 2017).

Though abuse or violence in childhood is frequently cited as a key factor that creates vulnerability for DMST, less is understood about the connections between violence in adolescence and DMST (Finkelhor et al., 2005; Twis, 2020; Twis et al., 2020). Previous studies have examined the complexities of romantic partner relationships that result in one partner trafficking the other and other work has explored the types of violence and abuse minors experience when being trafficked (Bejinariu et al., 2020; Doychak & Raghavan, 2020; Haney et al., 2020; Kellison et al., 2019; Roe-Sepowitz et al., 2017; Twis, 2020; Twis et al., 2020). However, to our knowledge, no empirical studies have investigated adolescent violence experiences that may exist beyond the DMST context nor the connections between these forms of interpersonal violence and a minor’s experiences of DMST. With the recognition that DMST is typically not the first form of abuse or violence minors have experienced, a polyvictimization framework has been proposed as a way to conceptualize DMST (Twis, 2020). The polyvictimization framework explores the context and nuance of DMST by placing it as one element of cumulative violence across the life course of youth at-risk of experiencing DMST and those who have experienced DMST (Twis, 2020). The polyvictimization framework highlights the value of exploring the context of DMST within a web of adolescent violence (such as IPV or teen dating violence) by noting that DMST is unlikely to be an isolated experience of abuse or violence in the lives of minors (Twis, 2020). Instead, experiences of DMST may co-occur with, be preceded by, or be followed by additional forms of violence in adolescence.

### Current Study

This study examined DMST in the polyvictimization framework by using a measure that captured minors engaging in commercial sex exchange. (Respondents were asked: “Have you ever given someone sex in exchange for drugs or money?” or “Since [the last interview], how many times have you given someone sex in exchange for drugs or money?”) The measured sex exchange was experienced by minor respondents in this study, meaning these minor sex exchange (MSE) experiences qualify as DMST under federal law.

Guided by the polyvictimization framework, the current study aimed to investigate violence victimization connections between DMST, measured as MSE, and other forms of interpersonal violence in adolescence by analyzing a large nationally representative sample of youth. The additional forms of violence victimizations considered in adolescence were IPV and community violence (Decker et al., 2018). The study’s research questions are as follows:

1. Is interpersonal violence victimization in adolescence associated with MSE?
2. Is adolescent community violence or IPV associated with MSE?
3. Does biological sex modify the relationship between interpersonal violence in adolescence and MSE?

## Methods

### Sample

This study analyzed data from the National Longitudinal Study of Adolescent to Adult Health (Add Health). Add Health is a large, nationally representative, longitudinal study with a sample of more than 20,000 adolescents in the United States who were in 7th-12th grade in 1994-1995 (Harris et al., 2009).

The analysis data set was comprised of information from the first two waves of Add Health, meaning the Waves I and II in-home interviews with the adolescents. Wave I included interview data with the original 20,743 respondents (10,480 females and 10,263 males) who were aged 12-20 in 1994-1995 (Harris, 2013). Wave II follow-up interviews with the respondents were collected from April to August 1996, and included 13,568 respondents (6,963 females and 6,605 males) who were aged 12-22 in 1996 (Harris et al., 2009). Thus, there was approximately one year between the Wave I and Wave II interviews. Note that Wave II interviews were not conducted with individuals who were high school seniors (12th grade) in Wave I unless they were part of the genetic sample.

To be eligible for inclusion in the analysis data set, interview data for each respondent must have been collected during both Waves I and II to support merging the datasets and include a nonzero Wave II sampling weight for each respondent. In addition, the respondents must have been 18-years-old or younger at Wave II, and have information available on the demographic variables of race/ethnicity, family structure, and age at Wave II.

### Measures

#### Demographic variables

*Biological sex*. For analysis purposes, biological sex was captured at Wave II when respondents were coded as either male or female.

*Race/ethnicity*. Race and ethnicity measures were created as a constructed variable from numerous interview questions and provided by Add Health researchers (Udry et al., 2003). This was included as a categorical variable with four options: Non-Hispanic White, Non-Hispanic Black, Hispanic, Other.

*Age.* Age at Wave II interview was included as a continuous variable.

*Highest parental education*. Highest parental education was used as a proxy variable for socioeconomic status, as recommended by other Add Health researchers (Kahn & Halpern, 2018). Constructed from multiple interview questions, it was included as a categorical variable with four options: no high school degree, high school graduate, some college attended, and college graduate or beyond.

*Family structure*. Family structure, previously constructed, reflects the type of family the respondent primarily resided with and was used as a categorical variable with four options: two biological parents, two parents (at least one of whom is nonbiological), single mother or single father, and other.

#### Adolescent risk behaviors

In order to account for certain adolescent behaviors, which may increase the risk of violence in adolescence, certain risk behaviors were controlled for in the multivariable analysis. These risk behavior controls were previously found to be associated with experiences of sex exchange in studies using Add Health data (Edwards et al., 2006; Kaestle, 2012; Ulloa et al., 2016):

*Binge drinking.* Respondents were asked about their risky alcohol use (Hayibor et al., 2019) at Waves I and II. Respondents indicated the frequency with which they engaged in binge drinking (five or more drinks in a row during one day) in the past 12 months. A drink was defined as more than a sip or a taste of someone else’s beer, wine, or liquor. Respondents who reported doing this two or more times each month were coded as engaging in binge drinking.

*Marijuana, cocaine, and other drug use*. Questions about marijuana use, cocaine use, and use of other illegal drugs (including LSD, PCP, ecstasy, mushrooms, speed, ice, heroin, or pills) and inhalants were asked during the Waves I and II interviews. If a respondent reported using one of these at any time, they were coded as having used that particular substance during their lifetime.

*Alcohol or drug use*. An overall measure of any risky alcohol or drug use was created using the alcohol and drug variables. If a respondent reported instances of binge drinking, marijuana use, cocaine use, or use of other illegal drugs or inhalants, they were coded as having experienced some type of alcohol or drug use.

*Shoplifting.* Shoplifting was measured at both Waves I and II by asking how often respondents took something from a store without paying for it. Respondents who reported shoplifting at least once at any time were coded as having ever shoplifted.

*Running away from home*. Running away was measured at both Waves I and II by asking how often respondents ran away from home. Respondents who reported having run away from home at least once at any time were coded as having ever run away from home.

#### Adolescent violence victimization

*Adolescent community violence victimization*. Community violence victimization was assessed at Waves I and II by asking respondents whether in the past 12 months someone pulled a knife or gun on them; shot, stabbed, or cut them; or jumped them. Respondents who answered yes to any of these questions were coded as having experienced community violence victimization.

*Adolescent intimate partner violence (IPV) victimization*. Add Health questions about IPV victimization were collected in the Wave II interview. Respondents were asked about this victimization for three types of partners: romantic partners, romantic interest partners, and nonromantic (potentially sexual) partners. (For more information on the designation of these categories, see the Introductory Guides of the Add Health Codebooks [Add Health Codebooks, 2020].) For each respondent experiencing such violence by one of these types of partners, respondents were asked whether the victimization was physical or psychological in nature. Physical violence victimization was assessed by asking whether the respondent’s partner had pushed or shoved them or thrown something at them that could hurt. Psychological violence victimization was assessed by asking whether the respondent’s partner had called them names, insulted them, treated them disrespectfully in front of others, swore at them, or threatened them with violence.

Responses to these questions were used to create six variables documenting respondents’ physical and psychological IPV experiences for each type of partner. These variables included the following: romantic partner violence that was physical; romantic partner violence that was psychological; romantic interest partner violence that was physical; romantic interest partner violence that was psychological; nonromantic partner violence that was physical; and nonromantic partner violence that was psychological. For respondents who reported multiple relationships with a particular type of partner (e.g., multiple romantic partners), these multiple partners were combined to create a composite measure of IPV experiences within that type of partnership. Two additional variables were created to document physical violence by any of the three types of partners and psychological violence by any of the three types of partners. A final variable was created representing either physical or psychological violence by any partner type (any IPV).

*Adolescent interpersonal violence*. Using information from the interview questions on community violence and IPV, we created an adolescent interpersonal violence victimization variable to indicate whether the respondent had ever experienced community or IPV.

#### Minor sex exchange (MSE)

*Sex exchange as a minor.* DMST was evaluated via a measure of MSE. MSE was assessed by asking respondents about their experiences of “giving someone sex in exchange for drugs or money,” an act that qualifies as DMST if the exchange happened when the respondent was a minor (Choi, 2015). Respondents at Wave I were asked if they had ever experienced MSE and at Wave II they were asked if they had experienced MSE between Waves I and II. Thus, when taken together, the Wave I and Wave II responses are a measure of MSE events prior to the Wave II interview.

In both the Wave I and Wave II interviews, respondents indicated the frequency of MSE events they had experienced (range: 0-834). Frequencies were summed across both interviews and presented descriptively. For analysis purposes, MSE responses were coded to reflect whether the respondent had ever exchanged sex for money or drugs either before the Wave I interview or between the time of the Wave I and II interviews.

### Analyses

Descriptive statistics for the key variables of interest were conducted for the whole analysis sample and separately for males and females. Bivariate cross-tabulations between MSE experiences and other forms of interpersonal violence victimization also were conducted on the entire sample and separately for males and females.

A multivariable logistic regression model was used to examine associations between community violence and IPV with MSE experiences. This model included data from the entire sample of males and females. More specifically, MSE (yes or no) was modeled as a function of demographic variables (biological sex, race/ethnicity, age, parental education, and family structure), adolescent risk behaviors (alcohol or drug use, shoplifting, running away), and interpersonal violence victimization (community violence, IPV). To assess sex differences in associations between MSE and the violence variables, as well as the risk behaviors, interaction terms were assessed between biological sex and each independent nondemographic variable.

Multiple imputation by chained equations (MICE) was conducted to account for item nonresponse on model variables that existed for a small portion (<5%) of the eligible sample. Data underwent 100 imputations (*m* = 100) using demographic variables (biological sex, race/ethnicity, age, family structure) as predictors. All analyses account for the complex survey design of Add Health—including weighting, stratification, and clustering—and the use of MICE (*m* = 100) in the application of respondent sampling weights (Wave II) and adjustment of variance estimates (Harris, 2013). Analyses were completed using Stata 16.1 (StataCorp, 2020).

## Results

The eligible Add Health sample size included 12,605 respondents (*n*_male_ = 6,071; *n*_female_ = 6,534). Of these eligible respondents, approximately 95% had no missing data for all included variables of interest (*n*_complete case_ = 12,015). The data for the remaining 5% who were eligible but missing responses on at least one variable of interest (*n*_partially complete_ = 590) underwent MICE (*m* = 100) to restore the analysis sample to the full, eligible size of 12,605 respondents.

### Demographic Characteristics

Table 1 presents descriptive findings on the demographic characteristics of the full sample, and by biological sex. The sample was approximately evenly split between males (48.16%) and females (51.84%). In the full sample, the majority of respondents identified as non-Hispanic White (65.91%) while 15.33% identified as non-Hispanic Black, 11.94% identified as Hispanic, and 6.82% identified by another race/ethnicity (Other). The average age at Wave II was 15.76 years. For highest parental education, 31.02% of respondents had a parent who had graduated college or beyond, followed by 29.97% with a parent who attended some college, 26.97% with a parent who had graduated high school, and 12.05% with a parent who had less than a complete high school education. Most respondents (55.03%) lived with two biological parents, while 17.15% lived with two parents, 23.78% lived with a single mother or a single father, and 4.04% lived in some alternative family structure.

### Risk Behaviors

As shown in Table 1, alcohol or drug use was the most common adolescent risk behavior (41.69%) with the most common substance use being marijuana (34.84%) followed by monthly binge drinking (17.08%), other illegal drugs or inhalants (14.92%), and cocaine (4.42%). Nearly one-third of respondents reported ever shoplifting (32.55%), and 11.33% of respondents indicated they had ever run away from home. When considering these risk behaviors by biological sex, a significantly larger percentage of males (44.21%) reported engaging in any alcohol or drug use than females (39.20%), and a greater percentage of males (20.81%) than females (13.41%) reported monthly binge drinking. While a significantly greater percentage of male respondents reported shoplifting (36.34%) than female respondents (28.80%), a higher percentage of females (12.40%) reported running away compared to males (10.25%).

### Violent Victimization

Table 1 shows that a sizeable portion (38.41%) of respondents experienced some form of interpersonal violence (IPV and/or community violence) in adolescence. The most common form of interpersonal violence victimization was community violence, which approximately one-fourth of respondents experienced in adolescence (25.40%). Community violence victimization was significantly more common for males (35.11%) than for females (15.80%). Approximately one in five respondents experienced IPV in adolescence (21.30%), and psychological IPV (19.64%) was more common than physical IPV (7.53%).

### MSE Experiences

The proportion of respondents who experienced MSE is included in Table 1, which reflects what has been published in other studies using Add Health data (Edwards et al., 2006; Kaestle, 2012; Ulloa et al., 2016). In the full sample, 3.53% of all respondents reported experiences of MSE, and a significantly greater proportion of male respondents (4.83%) reported having experienced MSE compared to females (2.26%).

**Table 1.** Descriptive Statistics for Demographics, Adolescent Risk Behaviors, Adolescent Violence Victimization, and MSE Experiences.

	All (*n =* 12,605)	Males (*n =* 6,071)	Females (*n =* 6,534)
	% (*SE*)	% (*SE*)	% (*SE*)
Demographics			
Race/ethnicity			
Hispanic	11.94 (1.59)	12.00 (1.64)	11.88 (1.64)
Non-Hispanic White	65.91 (2.80)	65.67 (2.85)	66.14 (2.87)
Non-Hispanic Black	15.33 (2.02)	14.84 (2.02)	15.81 (2.08)
Other	6.82 (0.77)	7.48 (0.91)	6.17 (0.75)
Age (Wave II) Mean (*SE*)	15.76 (0.10)	15.80* (0.10)	15.71* (0.11)
Highest parental education (SES)			
Less than high school graduation	12.05 (1.26)	11.98 (1.37)	12.12 (1.27)
High school graduate/GED	26.97 (1.13)	25.63 (1.25)	28.29 (1.25)
Some college or vocational education	29.97 (0.90)	31.05 (1.08)	28.89 (1.00)
College graduate or beyond	31.02 (1.78)	31.33 (1.88)	30.70 (1.85)
Family structure			
Two biological parents	55.03 (1.32)	55.23 (1.52)	54.84 (1.38)
Two parents	17.15 (0.49)	17.59 (0.63)	16.71 (0.64)
Single mother or single father	23.78 (1.12)	23.23 (1.27)	24.32 (1.19)
Other	4.04 (0.30)	3.95 (0.38)	4.13 (0.37)
Adolescent risk Behaviors			
Alcohol or drug use	41.69 (1.26)	44.21* (1.43)	39.20* (1.40)
Binge drinking (monthly prior 12 months)	17.08 (0.86)	20.81* (1.25)	13.41* (0.73)
Marijuana use (ever)	34.84 (1.30)	36.20 (1.42)	33.49 (1.53)
Cocaine use (ever)	4.42 (0.32)	4.77 (0.46)	4.07 (0.40)
Illegal drug (other) or inhalant use (ever)	14.92 (0.68)	14.76 (0.81)	15.09 (0.80)
Shoplifting	32.55 (0.83)	36.34* (0.98)	28.80* (0.99)
Running away from home	11.33 (0.47)	10.25* (0.58)	12.40* (0.62)
Adolescent violence victimization			
Any violence	38.41 (0.99)	44.84* (1.29)	32.07* (1.06)
Any community violence	25.40 (0.93)	35.11* (1.26)	15.80* (0.96)
Any partner violence (IPV)	21.30 (0.75)	20.84 (0.94)	21.75 (0.97)
Physical	7.53 (0.33)	7.14 (0.49)	7.93 (0.45)
Psychological	19.64 (0.74)	18.81 (0.88)	20.46 (0.97)
Minor sex exchange experiences			
Ever exchanged sex as a minor	3.53 (0.29)	4.83* (0.41)	2.26* (0.30)

*Note.* % (percentage) and *SE* (standard error) are weighted to be population-based estimates and account for multiple imputation.

* designates estimates significantly different for males vs. females (α = 0.05).

Table 2 displays the frequency of incidents of MSE events for the complete-case sample of males and females pooled and stratified by biological sex. For the complete-case sample, 3.12% of respondents (males: 4.19%; females: 2.09%) experienced one or more MSE incidents. The most common frequency was a single reported instance of sex exchanged for money or drugs (1.91% of all respondents).

**Table 2.** Frequency of MSE Experiences (Ever Having Exchanged Sex as Minor in Wave I and/or Wave II).

Frequency	All (*n =* 12,015)	Males (*n =* 5,726)	Females (*n =* 6,289)
	*n* (%)	*n* (%)	*n* (%)
0	11,633 (96.88)	5,476 (95.81)	6,157 (97.91)
1	242 (1.91)	147 (2.43)	95 (1.41)
2	38 (0.27)	28 (0.36)	10 (0.19)
3	19 (0.18)	* (0.17)	* (0.19)
4-6	26 (0.27)	15 (0.31)	11 (0.21)
7-19	26 (0.31)	* (0.39)	* (0.06)
20-834	31 (0.27)	* (0.41)	* (0.07)

*Note.* Counts and percentages reflect complete-case data (*n* = 12,015) prior to multiple imputation (where *n*_final_ = 12,605).

*n* values are raw counts; % (percentages) are weighted to be population-based estimates.

* = count suppressed due to small cell sizes.

### MSE and Violence Victimizations

Table 3 presents the bivariate frequencies of types of interpersonal violence victimization for respondents who experienced MSE and respondents who did not experience MSE. In general, greater percentages of minors with a history of sex exchange experienced interpersonal violence victimization (forms of IPV and/or community violence) compared to their peers without MSE experiences. More than half of the respondents who had experienced MSE also experienced community violence (51.09%), making community violence approximately twice as common among MSE survivors as their peers without MSE experiences (24.45%). For IPV, 30.44% of minors with sex exchange histories had a history of any IPV, while 20.96% of their non-MSE peers had experienced IPV. Among respondents who experienced MSE, psychological violence was the most common form of IPV (28.96%) and physical IPV (11.82%) was less common. Again, both these forms of IPV were less prevalent for respondents who had not experienced MSE (psychological: 19.30%; physical: 7.38%). These more prevalent victimization estimates of community violence and any IPV found in the full sample also existed for males and females when respondents were stratified by biological sex. For example, 53.81% of males and 45.37% of females who experienced MSE also experienced community violence compared to 34.16% of males and 15.12% of females without an MSE history. As reported in Table 3, the statistics for community violence significantly differed by biological sex, meaning that males who reported MSE had the highest percentage of community violence of the respondents, followed by females who reported MSE. Likewise, when examining IPV victimization across the three forms of IPV relationships/partnerships and the type of IPV (physical, psychological) experienced, nearly all estimates demonstrated a higher prevalence of IPV among respondents who experienced MSE than among their peers for both the full sample and the stratified male and female samples. The only exception to this pattern was for females who experienced IPV from a romantic partner. For physical IPV, psychological IPV, and any IPV in this partnership, female respondents who experienced MSE had a lower prevalence of IPV than their peers without an MSE history. This pattern for females who experienced IPV from a romantic partner likely accounted for the significant differences in biological sex for the romantic partner IPV categories of Table 3. Males and females were found to have significantly distinct estimates for any romantic partner IPV, physical IPV perpetrated by a romantic partner, and psychological IPV perpetrated by a romantic partner.

**Table 3.** Percentages and Standard Errors of Interpersonal Violence Victimizations During Adolescence Among MSE Youth vs. Non-MSE Youth.

	Youth Who Reported MSE (% = 3.53)			Youth Who Did Not Report MSE (% = 96.47)		
	All	Males (% = 67.87)	Females (% = 32.13)	All	Males (% = 49.02)	Females (% = 50.98)
	% (*SE*)	% (*SE*)	% (*SE*)	% (*SE*)	% (*SE*)	% (*SE*)
Any violence	60.87 (3.47)	62.37 (4.06)	57.68 (5.87)	37.59 (1.02)	43.95 (1.32)	31.48 (1.07)
Any community violence*	51.09 (3.48)	53.81 (4.06)	45.37 (6.51)	24.45 (0.93)	34.16 (1.27)	15.12 (0.97)
Any partner violence (IPV)	30.44 (3.01)	32.13 (3.49)	26.88 (4.30)	20.96 (0.75)	20.27 (0.95)	21.63 (0.97)
Physical	11.82 (2.19)	11.89 (2.67)	11.65 (3.07)	7.38 (0.33)	6.89 (0.50)	7.84 (0.45)
Psychological	28.96 (2.91)	30.41 (3.28)	25.90 (0.42)	19.30 (0.74)	18.22 (0.89)	20.34 (0.97)
Romantic partner violence*	4.87 (1.23)	6.87 (1.82)	0.65 (0.63)	1.66 (0.15)	1.81 (0.21)	1.52 (0.21)
Physical*	2.29 (0.93)	3.36 (1.40)	0.03 (0.03)	0.46 (0.08)	0.51 (0.12)	0.42 (0.10)
Psychological*	4.46 (1.18)	6.26 (1.76)	0.65 (0.63)	1.59 (0.14)	1.72 (0.20)	1.46 (0.21)
Romantic interest partner violence	21.94 (2.44)	20.75 (2.86)	24.44 (4.08)	18.96 (0.73)	17.81 (0.90)	20.06 (0.92)
Physical	8.42 (1.72)	7.28 (2.06)	10.82 (3.03)	6.61 (0.32)	6.01 (0.47)	7.19 (0.43)
Psychological	20.86 (2.36)	19.63 (2.67)	23.47 (4.01)	17.44 (0.72)	15.94 (0.84)	18.88 (0.91)
Nonromantic relationships (includes sexual) partner violence	7.16 (1.44)	9.01 (1.97)	3.25 (1.54)	1.65 (0.15)	2.10 (0.27)	1.23 (0.18)
Physical	1.69 (0.69)	2.11 (0.96)	0.80 (0.64)	0.53 (0.08)	0.61 (0.12)	0.46 (0.10)
Psychological	7.10 (1.43)	9.00 (1.97)	3.09 (1.53)	1.43 (0.15)	1.80 (0.26)	1.09 (0.18)

*Note.* % (percentage) and *SE* (standard error) are weighted to be population-based estimates and account for multiple imputation.

* designates violence categories in which the percentages across “Reported MSE” levels (youth who reported MSE vs. youth who did not report MSE) were significantly different for males vs. females (α = 0.05).

Table 4 presents results of logistic regression examining associations between MSE and experiences of community violence and IPV among respondents, with this analysis controlling for adolescent risk behaviors and demographic characteristics. Preliminary examinations investigated whether biological sex moderated the relationship between MSE and the nondemographic independent variables of the multivariable model (adolescent violence victimization; adolescent risk behaviors). Biological sex did not significantly (α = 0.05) modify any of the associations between interpersonal violence or risk behaviors and the odds of experiencing MSE, therefore stratified results for males and females are not presented in the multivariable model. Additionally, multiple IPV variables (e.g., physical IPV by a romantic partner, etc.) were included in preliminary models and were not significantly associated with MSE. As a result, more detailed measures of IPV were collapsed into a single composite variable for any IPV in multivariable regression model of Table 4.

**Table 4.** Results of Multivariable Model: Adjusted Odds Ratios for Any Minor Sex Exchange (MSE) (*n* = 12,605).

	MSE aOR (95% CI)
Adolescent violence victimization	
Any community violence	1.86* (1.32, 2.64)
Any partner violence (IPV)	1.14 (0.85, 1.54)
Adolescent risk behaviors	
Alcohol or drug use	1.87* (1.32, 2.65)
Shoplifting	1.11 (0.80, 1.54)
Running away from home	2.04* (1.53, 2.72)
Demographics	
Biological sex	
Male	1.00 (ref)
Female	0.50* (0.37, 0.68)
Race/ethnicity	
Non-Hispanic White	1.00 (ref)
Hispanic	0.66 (0.39, 1.14)
Non-Hispanic Black	1.86* (1.29, 2.68)
Other	0.87 (0.48, 1.57)
Age	0.98 (0.88, 1.10)
Highest parental education (SES)	
Less than high school graduation	2.50* (1.52, 4.11)
High school graduate/GED	2.53* (1.75, 3.66)
Some college or vocational education	1.92* (1.35, 2.73)
College graduate or beyond	1.00 (ref)
Family structure	
Two biological parents	1.00 (ref)
Two parents	0.64* (0.45, 0.92)
Single mother/single father	0.72 (0.51, 1.00)
Other	0.83 (0.44, 1.56)

*Note.* Adjusted odds ratio (aOR), (95% confidence interval).

*significant aOR.

(ref) = reference category.

Results show that community violence victimization was significantly and positively associated with MSE experiences (aOR: 1.86; 95% CI: 1.32-2.64). However, IPV victimization was not significantly associated with MSE (aOR: 1.14; 95% CI: 0.85-1.54). Alcohol or drug use was significantly positively associated with MSE (aOR: 1.87; 95% CI: 1.32-2.65), as was having run away from home (aOR: 2.04; 95% CI: 1.53-2.72). Females had significantly lower odds of experiencing MSE (aOR: 0.50; 95% CI: 0.37-0.68) compared to males. Non-Hispanic Black respondents had significantly greater odds of having experienced MSE (aOR: 1.86; 95% CI: 1.29-2.68) compared to non-Hispanic White respondents. Parental education was also significantly positively associated with MSE with all parental education categories compared to college graduate having elevated odds of experiencing MSE (Less than high school—aOR: 2.50; 95% CI: 1.52-4.11; High school graduate—aOR: 2.53; 95% CI: 1.75-3.66; Some college—aOR: 1.92; 95% CI: 1.35-2.73). For family structure, when compared to respondents living with two biological parents, those living with two parents (at least one nonbiological) had significantly decreased odds of MSE (aOR: 0.64; 95% CI: 0.45-0.92).

## Discussion

This study found a significant connection between MSE—a measure of DMST—and community violence experienced by adolescents. Despite the consistent bivariate pattern wherein a larger proportion of minors with a history of sex exchange experienced community violence and IPV compared to their non-MSE peers, IPV did not have a significant association with MSE in the multivariable model when controlling for demographic variables and adolescent risk behaviors. In this multivariable model, community violence, which captured whether the adolescent had been threatened or injured with a weapon or jumped, was significantly associated with whether a minor had ever given someone sex in exchange for money or drugs. Alcohol and drug use, as well as having run away from home, were also significantly associated with MSE. The associations between these forms of interpersonal violence or adolescent risk behaviors and DMST did not vary by biological sex, which suggests these connections between community violence victimizations and DMST experiences may not be meaningfully different for males and females.

This is the first study of which we are aware that links experiences of DMST to nonsexual interpersonal violence victimizations in adolescence using a large, population-based survey. These findings are consistent with other studies that have shown a connection between trafficking and other forms of violence, such as partner or domestic violence, and they reinforce the notion that DMST is not an isolated form of violence in the lives of minors (Koegler et al., 2020; Ortega-Senet & Tierney, 2020). This work also builds on prior Add Health analyses which found that DMST experiences were significantly linked to forced sex in adolescence, though it is unclear in these previous analyses whether respondents were considering their DMST experiences as forced sex or if the reported forced sex reflected one or more separate experiences from DMST (Edwards et al., 2006). Previous work has noted that DMST is not a “monolithic phenomenon” but an experience intersected with vulnerabilities and violence experiences of a minor’s life (Twis, 2020, p. 322). These findings shed light on how experiences of DMST may cluster with community violence victimizations, highlighting the interrelationship between these forms of violence (Finkelhor et al., 2005).

Females were found to be significantly protected against DMST, compared to males, in the multivariable regression results. Given that males are often overlooked or excluded from analyses focused on DMST experiences, these Add Health results are valuable in demonstrating the connections between DMST and community violence for both males and females, as the connection between DMST and community violence were not significantly distinct by biological sex (Moore et al., 2020; Robert & Willis, 2013). The protective relationship detected here for females may be due to the way in which the DMST measure was asked in Add Health. Previous work has noted that females may be more likely to report DMST or be identified as DMST survivors in contexts in which there is force, fraud, or coercion (via a third-party trafficker), or in which they consider their trafficker to be a romantic partner (Greeson et al., 2019; Reid, 2012). Males may be more likely to report DMST or be identified as DMST survivors in contexts in which sex is sold to meet survival needs or in which there is no third-party trafficker (Edinburgh et al., 2015; Mapp, 2016; Robert & Willis, 2013). The phrasing of the Add Health question reflecting DMST (which asked whether the respondent had ever given someone sex in exchange for money or drugs) may have caused the respondents to consider sex exchanges in which they had greater agency (i.e., in which there was no explicit force or coercion or no third-party trafficking) and resulted in more males than females reporting DMST experiences.

Additionally, the multivariable regression results connect a history of risky alcohol or drug use with DMST experiences, which is not surprising given the potential of the minor seeking drugs in exchange for sex built into the Add Health MSE interview question. Previous work has determined alcohol or drug use to be a significant predictor of DMST (Edwards et al., 2006; Klatt et al., 2014; Laird et al., 2020). Likewise, the relationship between running away from home and experiences of DMST have been noted by others. Running away has been noted as a significant risk factor for DMST, and minors who have run away may exchange sex to meet basic needs such as housing or food (Franchino-Olsen, 2019; IOM & NRC, 2013; Laird et al., 2020). In examining patterns of trafficking among youth who run away from foster care, Latzman and Gibbs (2020) noted that the relationship between trafficking and running away may be bidirectional, in which running away increases the risk of DMST and DMST increases the risk that a minor will run away from care. However, other research has indicated that the running away may sometimes reduce the risk of DMST by allowing the minor to leave an abusive or risky environment (Klatt et al., 2014). It seems the relationship between experiences of DMST and having run away from home is complex with the context and circumstances surrounding these events influencing the relationship between these experiences.

These findings reinforce the potential applicability of the polyvictimization framework—which focuses on the cumulative burden of violence—when considering the context of DMST in the lives of minors (Hamby et al., 2018; Twis, 2020). Though this analysis is unable to determine whether DMST preceded, co-occurred, or followed these reported community violence events, the results contextualize DMST as not an isolated incident of violence during adolescence. Rather, community violence is likely to be accompanied by experiences of DMST in adolescence, which provides a more nuanced understanding of the interconnected nature of victimizations. When considered alongside frequently cited risk factors of previous childhood abuse or maltreatment and sexual assault, these findings place DMST within a web of violence across the life course and speak to the need to carefully consider violence outside of DMST when responding to the needs of DMST survivors (Franchino-Olsen, 2019).

### Strengths and limitations

This study had the advantage of using a large, nationally representative, population-based survey to assess these rare and sensitive topics of violence in adolescence. The Add Health study design and sampling methods ensure that the sample was probability-based, which potentially expands the generalizability of the findings for adolescents in the United States. Additionally, by interviewing school-enrolled adolescents, many of whom may be typically overlooked as at-risk for DMST, the Add Health sample was potentially able to capture a greater portion of minors who had experienced one or more instances of DMST than would have been captured in a study focused on minors considered more at risk (e.g., homeless or runaway minors). This expanded sample of DMST survivors may have added nuance to this study’s findings. The findings also increase the diversity of our understanding around DMST due to the large number of males who were to be DMST survivors in the Add Health sample (67.87% of the MSE respondents were male).

The Add Health measure of MSE representing DMST cannot be used to distinguish between the natures of these DMST experiences. For example, the available variables do not provide insight into the context in which DMST events occurred. The measure is unable to determine if individuals exchanged sex for money or drugs to meet basic needs (such as food or housing), via coercion by a third-party, to meet a physiological need for drugs, or as a matter of choice in which they found it an appealing economic option to earn money/acquire drugs as a minor. Future work should seek to tease out how these distinct contexts for DMST affect associations between trafficking and interpersonal violence. The study is also limited by its diversity as minors who are not enrolled in school and who may be at even greater risk of DMST are not included in Add Health (Wolfe et al., 2018). Community violence was measured at Waves I and II, while IPV was assessed only at Wave II, making it difficult to determine if community violence was truly more prevalent than IPV for respondents across adolescence, as shown in our descriptive findings. Finally, Add Health at Wave I and Wave II does not offer any nuance around gender beyond binary biological sex, meaning gender identities that may experience a disproportionate risk of DMST—such as transgender, gender nonconforming, and gender nonbinary—could not be detected in this sample (Choi, 2015; Fedina et al., 2019; IOM & NRC, 2013).

### Implications

This study has implications for future research, policy, and practice for the antihuman trafficking field. Future research should further investigate the nature of the link between community violence and DMST to better understand the context of these intersecting forms of violence and whether minors experiencing these forms of violence have contact with key systems—such as education, child welfare, and criminal justice—in the time period surrounding these violent experiences. An improved understanding of the context and circumstances connecting DMST and community violence will deepen the field’s understanding of the events that lead to and follow DMST and should aid in the formation of empirical models or theories that can describe the links for DMST risk and protective factors to DMST experiences to subsequent outcomes.

These findings demonstrate the importance of trauma-informed, survivor-centered policy seeking to address DMST. The criminal justice, child welfare, and/or medical systems may interact with minors following episodes of community violence, running away from home, or alcohol or drug use. Given the connection to DMST for each of these circumstances, comprehensive policies should be implemented to ensure minors are screened for experiences of DMST and appropriate survivor-centered response protocols are in place for DMST disclosures to minimize harm and provide needed services.

Antitrafficking practice efforts should incorporate these findings to consider DMST as interconnected to other forms of violence, which can inform prevention and intervention efforts. In examining dynamics of DMST cases, Twis et al. (2020) highlighted the similarities found between the context and dynamics of the DMST violence and the dynamics known to exist in adolescent IPV or teen dating violence situations. The findings presented here linking DMST and community violence, along with the noted similarities between trafficker-inflicted violence and adolescent partner violence, emphasizes the need to integrate DMST prevention efforts in advocacy and programing that target community violence and/or IPV in adolescence (Twis, 2020; Twis et al., 2020). Recognizing the intersection of DMST and adolescent interpersonal violence, future DMST prevention and intervention strategies focused on education should provide messaging and resources for violence beyond DMST, while screening and/or service provisions for adolescents should ask about and incorporate resources for interpersonal violence along with DMST.

## Conclusion

This study fills important gaps in the antihuman trafficking field by contextualizing experiences of DMST in adolescence among community violence victimizations. Understanding that minors who experience community violence are also significantly more likely to experience DMST demonstrated the interconnected nature of trafficking violence to other forms of interpersonal violence. This connected nature should be considered by researchers and practitioners seeking to improve awareness of DMST and provide prevention or intervention efforts. Awareness efforts should be delivered with clear, trauma-informed messaging that addresses the additional forms of violence minors at risk of DMST or survivors of DMST have experienced. Prevention efforts should consider how violent environments or the connection between community violence and DMST can be used to reach high-risk minors and prevent DMST. Intervention work could also include screening for adolescent violence beyond DMST and providing care and services for needs that are tied to these additional violence victimizations. Future research should continue to explore these associations between adolescent violence and DMST and investigate how DMST survivors conceptualize these multiple forms of violence as potential harms and traumatic events.

## Author Biographies

**Hannabeth Franchino-Olsen**, PhD, MS, MPH, is a graduate of the Department of Maternal and Child Health at the University of North Carolina at Chapel Hill. Her research focuses on domestic and international issues of gender-based violence including adolescent intimate-partner violence and minor sex trafficking.

**Sandra L. Martin**, PhD, MSc, is a professor in the Department of Maternal and Child Health at the University of North Carolina at Chapel Hill. Dr Martin’s research includes secondary school education programs about sex trafficking, campus climate surveys of sexual misconduct, evaluation of sexual assault prevention, and work investigating differential responses/sequela after sexual assault.

**Carolyn T. Halpern**, PhD, is a professor and chair of the Department of Maternal and Child Health at the University of North Carolina at Chapel Hill. Dr Halpern’s research interests center on improving understanding of healthy sexual development and the implications of adolescent experiences for developmental and demographic processes into adulthood, particularly as these relate to sexual and romantic relationships.

**John S. Preisser**, PhD, is professor and chair of the Department of Biostatistics at the University of North Carolina at Chapel Hill. As deputy director of the biostatistics service with the North Carolina Translational & Clinical Sciences Institute (NC TraCS), Dr Preisser directs biostatistics collaboration, and he is a research fellow in the program on Aging, Disability, and Long-Term Care in the UNC Cecil G. Sheps Center for Health Services Research.

**Catherine Zimmer**, PhD, is a statistics consultant at the Odum Institute for Research in Social Science at the University of North Carolina at Chapel Hill. Dr Zimmer provides consultation to undergraduate students, graduate students, faculty, and staff on choosing appropriate analytical techniques and on how to apply them.

**Meghan Shanahan**, PhD, is an assistant professor in the Department of Maternal and Child Health at the University of North Carolina at Chapel Hill. Dr Shanahan’s research focuses on adverse events that potentially influence these trajectories and prevent children from realizing their full potential and includes examining prevention strategies to reduce child abuse and neglect.

## References

[bibr1-08862605211021967] Add Health Codebooks. (2020). *The national longitudinal study of adolescent to adult health*. Retrieved November 24, 2020, from https://addhealth.cpc.unc.edu/documentation/codebooks/

[bibr2-08862605211021967] Bejinariu A., Kennedy M. A., Cimino A. N., Bejinariu A., Kennedy M. A., & They A. N. C. (2020). “They said they were going to help us get through this …”: Documenting interactions between police and commercially sexually exploited youth. *Journal of Crime and Justice*. https://doi.org/10.1080/0735648X.2020.1807389

[bibr3-08862605211021967] Choi K. R. (2015). Risk factors for domestic minor sex trafficking in the United States. *Journal of Forensic Nursing*, 11(2), 66-76. https://doi.org/10.1097/JFN.0000000000000072 25996431 10.1097/JFN.0000000000000072

[bibr4-08862605211021967] Clawson H. J., Dutch N., Solomon A., & Grace L. G. (2009). *Human trafficking into and within the United States: A review of the literature*. U.S. Dept of Health and Human Services.

[bibr5-08862605211021967] Decker M. R., Wilcox H. C., Holliday C. N., & Webster D. W. (2018). An integrated public health approach to interpersonal violence and suicide prevention and response. *Public Health Reports*, 133(Suppl. 1), 65S-79S. https://doi.org/10.1177/0033354918800019 30426878 10.1177/0033354918800019 PMC6243443

[bibr6-08862605211021967] Doychak K., & Raghavan C. (2020). “No voice or vote”: Trauma-coerced attachment in victims of sex trafficking. *Journal of Human Trafficking*, 6(3), 339-357. https://doi.org/10.1080/23322705.2018.1518625

[bibr7-08862605211021967] Edinburgh L., Pape-Blabolil J., Harpin S. B., & Saewyc E. (2015). Assessing exploitation experiences of girls and boys seen at a child advocacy center. *Child Abuse & Neglect*, 46, 47-59. https://doi.org/10.1016/j.chiabu.2015.04.016 25982287 10.1016/j.chiabu.2015.04.016 PMC4760762

[bibr8-08862605211021967] Edwards J. M., Iritani B. J., & Hallfors D. D. (2006). Prevalence and correlates of exchanging sex for drugs or money among adolescents in the United States. *Sexually Transmitted Infections*, 82(5), 354-358. https://doi.org/10.1136/sti.2006.020693 16901917 10.1136/sti.2006.020693 PMC2563846

[bibr9-08862605211021967] Fedina L., Williamson C., & Perdue T. (2019). Risk factors for domestic child sex trafficking in the United States. *Journal of Interpersonal Violence*, 34(13), 2653-2673. https://doi.org/10.1177/0886260516662306 27470203 10.1177/0886260516662306

[bibr10-08862605211021967] Finkelhor D., Ormrod R., Turner H., & Hamby S. L. (2005). The victimization of children and youth: A comprehensive, national survey. *Child Maltreatment*, 10(1), 5-25. https://doi.org/10.1177/1077559504271287 15611323 10.1177/1077559504271287

[bibr11-08862605211021967] Franchino-Olsen H. (2019). Vulnerabilities relevant for commercial sexual exploitation of children/domestic minor sex trafficking: A systematic review of risk factors. *Trauma, Violence, and Abuse*, 22(1), 99-111. https://doi.org/10.1177/1524838018821956 10.1177/1524838018821956 30712473

[bibr12-08862605211021967] Franchino-Olsen H., Chesworth B. R., Boyle C., Rizo C. F., Martin S. L., Jordan B., Macy R. J., & Stevens L. (2020). The prevalence of sex trafficking of children and adolescents in the United States: A scoping review. *Trauma, Violence, and Abuse*. https://doi.org/10.1177/1524838020933873 10.1177/1524838020933873 PMC8685723 32588741

[bibr13-08862605211021967] Greenbaum V. J. (2014). Commercial sexual exploitation and sex trafficking of children in the United States. *Current Problems in Pediatric and Adolescent Health Care*, 44(9), 245-269. https://doi.org/10.1016/j.cppeds.2014.07.001 25131563 10.1016/j.cppeds.2014.07.001

[bibr14-08862605211021967] Greeson J. K. P., Treglia D., Wolfe D. S., & Wasch S. (2019). Prevalence and correlates of sex trafficking among homeless and runaway youths presenting for shelter services. *Social Work Research*, 43(2), 91-100. https://doi.org/10.1093/swr/svz001

[bibr15-08862605211021967] Hamby S., Taylor E., Jones L., Mitchell K. J., Turner H. A., & Newlin C. (2018). From poly-victimization to poly-strengths: Understanding the web of violence can transform research on youth violence and illuminate the path to prevention and resilience. *Journal of Interpersonal Violence*, 33(5), 719-739. https://doi.org/10.1177/0886260517744847 29411696 10.1177/0886260517744847

[bibr16-08862605211021967] Haney K., Lebeau K., Bodner S., Czizik A., Young E., & Hart M. (2020). Sex trafficking in the United States: A scoping review. *Journal of Evidence-Based Social Work*, 17(6), 714-748. https://doi.org/10.1080/26408066.2020.1765934 32678726 10.1080/26408066.2020.1765934

[bibr17-08862605211021967] Harris K. M. (2013). *The add health study: Design and accomplishments*. https://addhealth.cpc.unc.edu/wp-content/uploads/docs/documentations/guides/DesignPaperWave_I-IV.pdf

[bibr18-08862605211021967] Harris K. M., Halpern C. T., Whitsel E., Hussey J., Tabor J., Entzel P., & Udry J. R. (2009). *The national longitudinal study of adolescent to adult health: Research design*. Retrieved July 2, 2019, from https://www.cpc.unc.edu/projects/addhealth/design 10.1093/ije/dyz115 PMC6857761 31257425

[bibr19-08862605211021967] Hayibor L. A., Zhang J., & Duncan A. (2019). Association of binge drinking in adolescence and early adulthood with high blood pressure: Findings from the national longitudinal study of adolescent to adult health (1994-2008). *Journal of Epidemiology and Community Health*, 73(7), 652-659. https://doi.org/10.1136/jech-2018-211594 30971421 10.1136/jech-2018-211594

[bibr20-08862605211021967] IOM NRC. (2013). Risk factors for and consequences of commercial sexual exploitation and sex trafficking of minors. In Clayton E. W., Krugman R. D., & Simon P. (Eds.), *Confronting commercial sexual exploitation and sex trafficking of minors in the United States* (p. 90). https://doi.org/10.17226/18969

[bibr21-08862605211021967] Kaestle C. E. (2012). Selling and buying sex: A longitudinal study of risk and protective factors in adolescence. *Prevention Science*, 13(3), 314-322. https://doi.org/10.1007/s11121-011-0268-8 22350114 10.1007/s11121-011-0268-8

[bibr22-08862605211021967] Kahn N. F., & Halpern C. T. (2018). The relationship between cognitive ability and experiences of vaginal, oral, and anal sex in the United States. *Journal of Sex Research*, 55(1), 99-105. https://doi.org/10.1080/00224499.2016.1247149 27897438 10.1080/00224499.2016.1247149 PMC6628929

[bibr23-08862605211021967] Kellison B., Torres M. I. M., Kammer-Kerwick M., Hairston D., Talley M., & Busch-Armendariz N. (2019). *“To the public, nothing was wrong with me” life experiences of minors and youth in Texas at risk for commercial sexual exploitation*. The University of Texas at Austin.

[bibr24-08862605211021967] Klatt T., Cavner D., & Egan V. (2014). Rationalising predictors of child sexual exploitation and sex-trading. *Child Abuse and Neglect*, 38(2), 252-260. https://doi.org/10.1016/j.chiabu.2013.08.019 24070694 10.1016/j.chiabu.2013.08.019

[bibr25-08862605211021967] Koegler E., Howland W., Gibbons P., Teti M., & Stoklosa H. (2020). “When her visa expired, the family refused to renew it,” Intersections of human trafficking and domestic violence: Qualitative document analysis of case examples from a major midwest city. *Journal of Interpersonal Violence*. https://doi.org/10.1177/0886260520957978 10.1177/0886260520957978 32924747

[bibr26-08862605211021967] Kotrla K. (2010). Domestic minor sex trafficking in the United States. *Social Work*, 55(2), 181-187. https://doi.org/10.1093/sw/55.2.181 20408359 10.1093/sw/55.2.181

[bibr27-08862605211021967] Laird J. J., Klettke B., Hall K., Clancy E., & Hallford D. (2020). Demographic and psychosocial factors associated with child sexual exploitation: A systematic review and meta-analysis. *JAMA Network Open*, 3(9), e2017682. https://doi.org/10.1001/jamanetworkopen.2020.17682 32960280 10.1001/jamanetworkopen.2020.17682 PMC7509625

[bibr28-08862605211021967] Latzman N. E., & Gibbs D. A. (2020). *Examining the link: Foster care runaway episodes and human trafficking*. U.S. Department of Health & Human Services.

[bibr29-08862605211021967] Mapp S. C. (2016). *Domestic minor sex trafficking* (1st ed.). Oxford University Press.

[bibr30-08862605211021967] Moore J., Fitzgerald M., Owens T., Slingsby B., Barron C., & Goldberg A. (2020). Domestic minor sex trafficking: A case series of male pediatric patients. *Journal of Interpersonal Violence*. https://doi.org/10.1177/0886260519900323 10.1177/0886260519900323 31948332

[bibr31-08862605211021967] National Academies of Sciences Engineering and Medicine. (2020). *Estimating the prevalence of human trafficking in the United States: Considerations and complexities: Proceedings of a workshop*. The National Academies Press. https://doi.org/10.17226/25614

[bibr32-08862605211021967] Ortega-Senet M. B., & Tierney E. M. (2020). Critical knots, tensions, and daily resistances in the work against commercial sexual exploitation of children: A reflection from Chilean practitioners. *International Social Work*. https://doi.org/10.1177/0020872819899434

[bibr33-08862605211021967] Reid J. A. (2012). Exploratory review of route-specific, gendered, and age-graded dynamics of exploitation: Applying life course theory to victimization in sex trafficking in North America. *Aggression and Violent Behavior*, 17(3), 257-271. https://doi.org/10.1016/j.avb.2012.02.005

[bibr34-08862605211021967] Robert N., & Willis B. (2013). *And boys too*. https://www.ecpatusa.org/ecpat-reports/

[bibr35-08862605211021967] Roe-Sepowitz D., Gallagher J., Hogan K., Ward T., Denecour N., & Bracy K. (2017). *A six-year analysis of sex traffickers of minors: Exploring characteristics and sex trafficking patterns*. https://socialwork.asu.edu/sites/default/files/ASU-Sex-Traffickers-of-Minors-Six-Year-Study-Full-Report-April-2017.pdf

[bibr36-08862605211021967] StataCorp. (2020). *Stata statistical software: Release 16.1*. StataCorp LP.

[bibr37-08862605211021967] Stransky M., & Finkelhor D. (2008). *How many juveniles are involved in prostitution in the U.S.?* (Vol. 03824). http://www.unh.edu/ccrc/prostitution/Juvenile_Prostitution_factsheet.pdf

[bibr38-08862605211021967] Twis M. K. (2020). Risk factor patterns in domestic minor sex trafficking relationships. *Journal of Human Trafficking*, 6(3), 309-326. https://doi.org/10.1080/23322705.2019.1627775

[bibr39-08862605211021967] Twis M. K., Gillespie L., & Greenwood D. (2020). An analysis of romantic partnership dynamics in domestic minor sex trafficking case files. *Journal of Interpersonal Violence*. https://doi.org/10.1177/0886260520960302 10.1177/0886260520960302 32975466

[bibr40-08862605211021967] Udry J. R., Li R. M., & Hendrickson-Smith J. (2003). Health and behavior risks of adolescents with mixed-race identity. *American Journal of Public Health*, 93(1), 1865-1870. 14600054 10.2105/ajph.93.11.1865 PMC1448064

[bibr41-08862605211021967] Ulloa E., Salazar M., & Monjaras L. (2016). Prevalence and correlates of sex exchange among a nationally representative sample of adolescents and young adults. *Journal of Child Sexual Abuse: Research, Treatment, & Program Innovations for Victims, Survivors, & Offenders*, 25(5), 524-537. https://doi.org/10.1080/10538712.2016.1167802 10.1080/10538712.2016.1167802 PMC5613935 27266400

[bibr42-08862605211021967] Department of Health U.S., Youth Human Services Administration for Children, & (ACYF). Families (2013). *Guidance to states and services on addressing human trafficking of children and youth in the United States*. https://www.acf.hhs.gov/cb/resource/human-trafficking-guidance

[bibr43-08862605211021967] Victims of Trafficking and Violence Protection Act. (2000). Public Law 106–386—Oct. 28, 2000. https://www.govinfo.gov/content/pkg/PLAW-106publ386/pdf/PLAW-106publ386.pdf

[bibr44-08862605211021967] Wolfe D. S., Greeson J. K. P., Wasch S., & Treglia D. (2018). *Human trafficking prevalence and child welfare risk factors among homeless youth a multi-city study*. The Field Center for Children’s Policy, Practice & Research. https://calio.dspacedirect.org/handle/11212/3799

